# GLP1 and GIP are involved in the action of synbiotics in broiler chickens

**DOI:** 10.1186/s40104-017-0227-8

**Published:** 2018-01-26

**Authors:** Pawel Antoni Kolodziejski, Maciej Sassek, Daniela Chalupka, Natalia Leciejewska, Leszek Nogowski, Pawel Mackowiak, Damian Jozefiak, Katarzyna Stadnicka, Maria Siwek, Marek Bednarczyk, Tomasz Szwaczkowski, Ewa Pruszynska-Oszmalek

**Affiliations:** 1 Department of Animal Physiology and Biochemistry, Poznan University of Life Sciences, Wolynska 35, 60-637 Poznan, Poland; 2 Department of Animal Nutrition and Feed Management, Poznan University of Life Sciences, Wolynska 33, 60-637 Poznan, Poland; 3 Department of Genetics and Animal Breeding, Poznan University of Life Sciences, Wolynska 33, 60-637 Poznan, Poland; 4 Department of Animal Biochemistry and Biotechnology, UTP University of Science and Technology, 85-084 Bydgoszcz, Poland

**Keywords:** GIP, GLP-1, Incretins, In ovo, Synbiotics

## Abstract

**Background:**

In order to discover new strategies to replace antibiotics in the post-antibiotic era in meat-type chicken production, two new synbiotics were tested: (*Lactobacillus salivarius* IBB3154 plus galactooligosaccharide (Syn1) and *Lactobacillus plantarum* IBB3036 plus raffinose family oligosaccharides (Syn2).

**Methods:**

The synbiotics were administered via syringe, using a special automatic system, into the egg air chamber of Cobb 500 broiler chicks on the 12^th^ day of egg incubation (2 mg of prebiotics + 10^5^ cfu bacteria per egg). Hatched roosters (total 2,400) were reared on an experimental farm, kept in pens (75 animals per pen), with free access to feed and water. After 42 d animals were slaughtered. Blood serum, pancreas, duodenum and duodenum content were collected.

**Results:**

Syn2 increased trypsin activity by 2.5-fold in the pancreas and 1.5-fold in the duodenal content. In the duodenum content, Syn2 resulted in ca 30% elevation in lipase activity and 70% reduction in amylase activity. Syn1 and Syn2 strongly decreased expression of mRNA for GLP-1 and GIP in the duodenum and for GLP-1 receptors in the pancreas. Simultaneously, concentrations of the incretins significantly diminished in the blood serum (*P* < 0.05). The decreased expression of incretins coincides with changed activity of digestive enzymes in the pancreas and in the duodenal content. The results indicate that incretins are involved in the action of Syn1 and Syn2 or that they may even be their target. No changes were observed in key hormones regulating metabolism (insulin, glucagon, corticosterone, thyroid hormones, and leptin) or in metabolic indices (glucose, NEFA, triglycerides, cholesterol). Additionally, synbiotics did not cause significant changes in the activities of alanine and aspartate aminotransferases in broiler chickens. Simultaneously, the activity of alkaline phosphatase and gamma glutamyl transferase diminished after Syn2 and Syn1, respectively.

**Conclusion:**

The selected synbiotics may be used as in ovo additives for broiler chickens, and Syn2 seems to improve their potential digestive proteolytic and lipolytic ability. Our results suggest that synbiotics can be directly or indirectly involved in incretin secretion and reception.

## Background

In recent years the administration of natural non-antibiotic and non-hormonal growth stimulators supporting chicken health has been most intensively studied. Prebiotics and probiotics, either alone or in combination (synbiotics), are considered to be a helpful alternative in poultry breeding to replace the usage of antibiotics as growth promoters banned in some parts of the world, strengthen health, improve production parameters and diminish environmental pollution. Both the manner and moment of addition of biologically active compounds seem to be important to achieve the best effects. The in ovo technique applied in the present paper is a relatively new method of supplementation, which allows for the application of prebiotics or synbiotics in early developmental stages and enables the modulation of gastrointestinal tract activity [1, 2]. Besides being an important location for bacterial settlement and enzymatic digestion of feed, the intestine is also a source of incretins which play a significant role, among others, in the regulation of pancreatic function.

Synbiotics may contribute to the modification of the gut activity. Some results indicate that synbiotics can affect incretins in humans and rodents [3–5]. Moreover, previous research has shown that synbiotics are able to modify the entire spectrum of phenotypic features, e.g. growth, intestinal tissue structure, pancreas potency, molecular changes in liver, and also spleen, tonsils and caecal bacterial populations and caecal fermentation [6, 7]. There are no reports related to the impact of synbiotics on incretins in poultry. Therefore, in birds treated with synbiotics, we decided to study simultaneously the activity of digestive enzymes in the pancreas and duodenum, and the synthesis, secretion and reception of two important incretins –– gastric inhibitory polypeptide or glucose-dependent insulinotropic peptide (GIP) and glucagon-like peptide 1 (GLP-1) –– exerting stimulatory action on insulin secretion. GIP, described by Brown et al. [8], is a 42-amino acid compound synthesized and secreted by K cells in the entire small intestine. The biological activity of GIP is regulated via gastric inhibitory peptide receptors (GIP-R). GIP-R in humans and rodents are expressed in various tissues and organs, such as the brain, pancreas, small intestine, stomach, adipose tissue, pituitary, heart, spleen, thymus, lung, kidney, and thyroid [9–11]. Among the various functions of GIP the following can be mentioned: reduction of gastric acid secretion [9]; stimulation of insulin secretion [9, 12]; control of food intake as a negative regulator of NPY [9]; stimulation of lipogenesis in fat tissue [13]; and a positive effect on bone formation and downregulation of bone resorption [14]. Unfortunately, the biological functions of GIP in chicken are still insufficiently understood. Equally important for the regulation of the body’s metabolism is another incretin – glucagon-like peptide 1 (GLP-1). GLP-1 is secreted by L-cells located mainly in the duodenum, ileum, and colon [15]. There are two biologically active forms of GLP-1: GLP-1-(7–37) and GLP-1-(7–36)NH_2_, and both derive from the proglucagon molecule by post-translational processing [16–18]. GLP-1 inhibits gastric emptying [9], glucose production in the liver [19], peristaltic movements, and pancreas functions [18, 20], and decreases appetite [21]. GLP-1R (glucagon-like peptide 1 receptor) belongs to the group of G-protein coupled receptors and is expressed in various tissues, such as the central nervous system, pancreatic islets, pancreas, stomach, intestine, liver, and fat tissue [22–24].

Considering that the final action of hormones is the result of their synthesis, secretion and reception, the present paper studied the expression of mRNA for GIP and GLP-1 in the duodenum, for their receptors in the pancreas, and the levels of both incretins in the blood serum. Previously, we have demonstrated the effect on the enzymatic activity of the pancreas of in ovo administered synbiotics composed of inulin plus *Lactococcus lactis* subsp. *lactis*, and of Bi^2^tos plus *Lactococcus lactis* subsp. *cremoris* [1]. In the present study, two synbiotics were chosen (Syn1 – *Lactobacillus salivarius* IBB3154 plus galactooligosaccharide [Bi^2^tos]; Syn2 – *Lactobacillus plantarum* IBB3036 plus raffinose family oligosaccharides [RFO]) and, besides the enzymatic activity of the pancreas, analyses also focused on enzymatic activity in the duodenum content. Moreover, a wide panel of hormones regulating metabolism (insulin, glucagon, corticosterone, leptin, and thyroid hormones), as well as a broad spectrum of biochemical indices (glucose, non-esterified fatty acids, triglycerides, total and free cholesterol, total proteins and albumins) and diagnostic enzymes (alanine aminotransferase, aspartate aminotransferase, alkaline phosphatase, and gamma glutamyl transferase) were analyzed. All the analyses allowed us to answer the question of whether the chosen in ovo implemented synbiotics act long-term and whether they modulate the hormonal and enzymatic activities of the digestive tract.

## Methods

This study was undertaken with the approval of the Polish Local Ethical Commission (Bydgoszcz, Poland, No. 36/2012).

### Experimental design

Selection of synbiotics was performed based on the in vitro and in vivo experiments described by Dunislawska et al. [25]. Also, detailed procedures for the experimental setup and rearing conditions were presented previously by Dunislawska et al. [25]. In brief, 5,850 eggs (approx. 65 g each) from Cobb500FF hens (42-week-old) were incubated at a commercial hatchery. On d 12, eggs were randomly allotted to 3 groups and injected with 0.2 mL of either saline or synbiotics. The control group received pure saline (0.9%), whereas experimental groups received synbiotics: Syn1 – *Lactobacillus salivarius* IBB3154 plus galactooligosaccharide [Bi^2^tos, Clasado Biosciences, Ltd., Jersey, UK]; Syn2 – *Lactobacillus plantarum* IBB3036 plus raffinose family oligosaccharides [RFO – combination of 6.1% of sucrose, 9.4% of raffinose, 65.2% of stachyose, 18.0% of verbascose, and 1.3%.of other saccharides] [26, 27]. The prebiotic component was given at a dose of 2 mg and bacteria at a quantity of 10^5^ cfu per egg.

For further investigations only those roosters were qualified which had hatched from eggs previously in ovo injected into air chamber eggs (total 2400). The roosters were reared on a commercial farm registered also as an experimental farm (Piast, Olszowa, Poland). Birds from control and experimental groups were kept on the floor in pens (75 animals per pen) and had free access to feed and water. Body weight, feed consumption, feed conversion efficiency and mortality were measured. The rearing data from the experiment are described by Dunislawska et al. [25]. For the investigations described in the paper, 42-day-old birds randomly chosen from the pens were used.

### Blood collection

On d 42 birds were slaughtered, the blood was collected and then the blood serum obtained. Immediately after the blood collection, pancreases and duodenum were excised. Also, the duodenum content was taken for analyses. Next, the whole material was frozen and kept for further investigations at −80 °C.

### Pancreatic enzyme activity

The activity of pancreatic enzymes was measured as described previously [1].

In brief, after being weighed pancreases were homogenized in TBS buffer on ice and 20% homogenates were centrifuged (10,000×*g* for 16 min.). Activities of amylase, lipase, and trypsin were measured using appropriate commercial colorimetric tests (Biovision, USA). For measurement of lipase activity, homogenates were diluted 100× using TBS buffer, while for measurement of amylase activity these were diluted 1,000X with commercially supplied buffer. In the case of trypsin measurement, diluted supernatants (100× with TBS) were incubated with 1% enterokinase (prepared in 0.1 mol/L Tris-HCl with 0.1 mol/L CaCl_2_; pH 7.2). The temperatures for measurement of enzyme activities were as follows: for amylase and trypsin 25 °C, and for lipase 37 °C. Activities were measured as the amount of product formed during reactions (glycerol, nitrophenol, *p*-nitroaniline) and these are expressed in the manuscript as % of control.

To determine the activity of the lipase, amylase and trypsin in the duodenal content, 250 μL of PBS was added to 100 mg of content, the mixture was homogenized on ice and centrifuged (10,000×*g* for 16 min.). The activity of enzymes was measured using the commercial kits mentioned above.

### Hormonal profile

The concentrations of blood serum hormones involved in the regulation of feed intake and catabolic/anabolic pathways and carbohydrate/lipid/protein turnover were investigated in the blood serum as follows using commercial RIA and EIA kits: insulin (Insulin RIA kit, Millipore, USA), total thyroxine (Thyroxine (T4) kit, RIA – Cis International), free thyroxine (Free Thyroxine (fT4) kit, RIA – Cis International), total triiodothyronine (Triiodothyronine (T3) kit, RIA – Cis International), free triiodothyronine (Free Triiodothyronine (fT3) kit, RIA – Cis International), corticosterone (Corticosterone ELISA kit – Enzo Life Sci., Warsaw, Poland), leptin (Multi-Species Leptin RIA, Millipore, USA), incretins GIP (Gastric Inhibitory Polypeptide) (SunRed, Jufengyuan Road, Baoshan, District, Shanghai), and GLP-1 (Glucagon-like Polypeptide-1) (Phoenix Pharmaceuticals Inc., USA). In order to verify the specificity of binding of kits, serum dilution curve was performed. For this purpose serum from five randomly chickens was diluted 2×, 4× and 10 times in Elisa/RIA buffer, and next concentrations of investigated hormones were determined in all dilutions.

### Enzyme activity in the blood serum

The activity of diagnostic enzymes and concentration of main blood biochemical parameters were analyzed in the blood serum. The activities of alanine (ALT; GPT) and aspartate (AST; GOT) aminotransferases as well as alkaline phosphatase (ALP) and gamma glutamyl transpeptidase (GGT) were estimated using commercial kits (Pointe Scientific, USA).

### Biochemical parameters in the blood serum

The parameters of carbohydrate (glucose), protein (total proteins and albumins), and lipid (triglycerides, free fatty acids, cholesterol) metabolism were estimated: glucose was measured using a Glucose Assay Kit – Pointe Scientific (USA), total protein and albumins were estimated using a Total Proteins Kit (Alpha Diagnostics, Poland) and Total Albumins Kit (Alpha Diagnostics, Poland), triglycerides were measured using a kit from Pointe Scientific (USA) (Alpha Diagnostics, Poland), free fatty acids were estimated with a kit from Wako Chemicals (USA), and cholesterol was measured with a Cholesterol/Cholesteryl Ester Quantitation Kit – BioVision (USA).

### Real-Time PCR

Determination of incretins (*GIP*; *GLP-1*) and their receptors (*GIP-R*; *GLP-1R*) mRNA and incretins serum concentration is described below. Isolation of total RNA was performed using Tripure reagent (Roche, Germany) according to the manufacturer’s instructions. The efficiency of isolation and the quality of isolated RNA were determined using NanoDrop 1000. Additionally, the integrity of RNA was determined by electrophoresis in 1% agarose gel. cDNA was synthesized from 1 μg of total RNA using a high-capacity cDNA reverse transcription kit (Thermo Fisher Scientific). Moreover, to exclude contamination of genomic DNA, we performed all RT-PCRs in parallel without added RT and detected no signals. Primers for reactions were designed using Primer-BLAST (Primers sequences are presented in Table 1). Real-Time PCR was performed using QuantStudio 12 K Flex™ Real-Time PCR and Fast SYBR Green Master Mix (Life Technologies, Grand Island, NY, USA). Amplification involved one cycle at 95 °C for 1 min for initial denaturation and then 45 cycles consisting of denaturation (95 °C for 3 s) and annealing (62 °C for 30 s). Detection of the fluorescent product was set at the last step of each cycle. To determine the specificity of amplification, analyses of product melting were performed after each amplification (0.1 °C/s increment from 65 °C to 95 °C, with fluorescence collection at 0.1 °C intervals). Relative gene expression was evaluated by Delta Delta CT (ΔΔCT) with *GAPDH* as a reference.

**Table 1:** Polymerase chain reaction primer sequences and product size

Genes	Forward primer (5′→3′)	Reverse primer (5′→3′)	Product Size, bp
*GLP-1*	CCAAGCGTCATTCTGAATTTG	TGACCTTCCAAATAAGAGGTGATA	76
*GIP*	CGCAGTGAGTGACCAAAGC	TAGGAGCCATGCAAGGAAGT	67
*GLP-1R*	GTGTACCGGTTCTGCACCTC	GGGCAGAGTCGAGTTCTCCT	60
*GIP-R*	GCGTTACCTCTACGAGAACGA	GCGGATGATCCACCACAC	70
*GAPDH*	GTGAAAGTCGGAGTCAACGG	ACAGTGCCCTTGAAGTGTCC	170

### Statistical analysis

Statistical analyses were performed as previously described [1]. In brief, all data were analyzed using one-way ANOVA followed by the Duncan’s multiple range test. Data are presented as means ±SEM, (*n* = 8 per group) and *P* < 0.05 was considered statistically significant. Statistical significance compared to controls was marked * *P* < 0.05, ** *P* < 0.01.

## Results

### Synbiotics modulate *GLP-1*/*GLP-1R* and *GIP*/*GIP-R* mRNA expression levels and decrease concentrations of GLP-1 and GIP in the blood serum

**Fig. 1:**
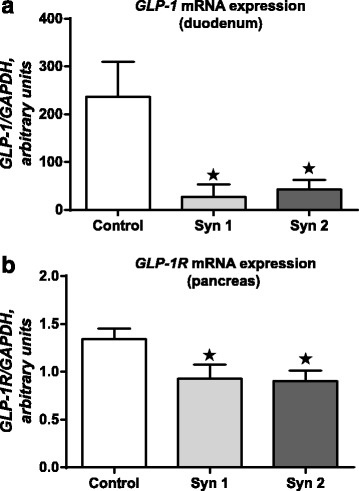
*GLP-1* and *GLP-1R* mRNA expression in chicken duodenum and pancreas. Results are means ± SEM (*n* = 8). *P < 0.05, **P < 0.01 compared with control

**Fig. 2:**
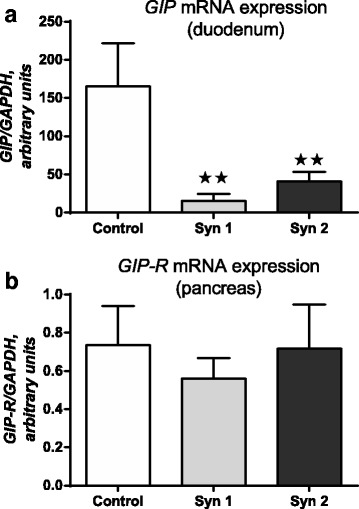
*GIP* and *GIP-R* mRNA expression in chicken duodenum and pancreas. Results are means ± SEM (n = 8). *P < 0.05, **P < 0.01 compared with control

**Fig. 3:**
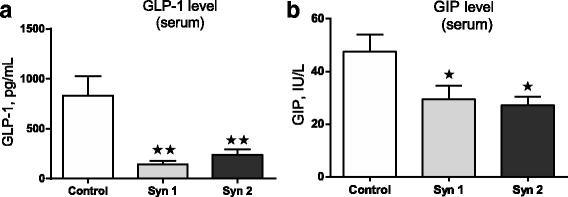
GLP-1 and GIP concentrations in blood serum. Results are means ± SEM (n = 8). *P < 0.05, **P < 0.01 compared with control

In the experiment, the effect of synbiotics on the expression of mRNA for GLP-1, GIP and their receptors mRNA in chicken duodenum and pancreas was stated. In the duodenum, downregulation of *GLP-1* mRNA level (Fig. 1a) was observed after Syn1 (*P* < 0.05) and Syn2 (*P* < 0.05). Similar results were obtained for *GIP*. Syn1 (*P* < 0.01) and Syn2 (*P* < 0.01) decreased its mRNA expression (Fig. 2a). Also, we investigated the effects of synbiotics on *GIP-R* and *GLP-1R* mRNA in the chicken pancreas. In synbiotic groups, we found lower mRNA levels of *GLP-1R* compared to the control group (Fig. 1b; *P* < 0.05). Statistically significant differences were not observed for *GIP-R* mRNA in the pancreas (Fig. 2b). Simultaneously, the effect of the synbiotics on serum levels of GIP and GLP-1 was examined and, using immunoenzymatic assays, lower concentrations of the both incretins were found. The effect of synbiotics on GLP-1 concentration (*P* < 0.01) was more pronounced; however, statistically significant changes were also observed in GIP levels (*P* < 0.05) (Fig. 3).

### Synbiotics modulate pancreatic enzyme activity

The decreased expression of incretins coincided with changed activity of digestive enzymes in the pancreas and in the duodenal content. The activity of all investigated enzymes in the pancreas was elevated; however, a statistically significant difference was observed only for trypsin activity in birds which had been administered Syn2. On the other hand, in the duodenal content activity of trypsin and lipase were noticeably heightened after synbiotics with significant changes observed after Syn2. In turn, amylase activity in the duodenum was less than half that of the control value and again a statistically significant change was observed only for Syn2. All alterations are included in Table 2.

**Table 2:** Changes in amylase, lipase and trypsin activities in pancreas and duodenum content after in ovo synbiotic treatment

Enzymes	Determination location	Control	Synbiotic 1	Synbiotic 2
Trypsin	Pancreas	100.0 ± 38.52	191.49 ± 25.45	246.49* ± 25.13
	Duodenum content	100.0 ± 18.39	93.99 ± 27.35	150.69* ± 14.55
Lipase	Pancreas	100.0 ± 27.43	158.43 ± 30.94	128.96 ± 32.98
	Duodenum content	100.0 ± 6.41	110.58 ± 17.86	127.43* ± 10.66
Amylase	Pancreas	100.0 ± 27.03	117.94 ± 18.46	116.64 ± 12.79
	Duodenum content	100.0 ± 26.21	48.07 ± 20.78	30.05* ± 7.63

Differences are expressed as % of control. Results are the mean ± SEM (n = 8). *P < 0.05

### Effect of synbiotics on biochemical parameters and enzyme activity in blood serum

The concentrations of other blood parameters including hormones are presented in Table 3. Besides the changes noticed for incretins and their receptors and for the activity of digestive enzymes, no significant changes were stated for the measured hormones or biochemical compounds. The levels of insulin, glucagon, corticosterone, thyroid hormones, and leptin were similar in both control and experimental groups. Moreover, the insulin/glucagon molar ratio was not evidently changed and no significant alterations in metabolic parameters were shown. The levels of serum glucose, non-esterified fatty acids, triglycerides, cholesterol, total protein and albumins did not change. The synbiotics used did not cause bigger changes in the activities of two diagnostic enzymes: alanine and aspartate aminotransferases. However, the activity of ALP and GGT markers was modified. Alkaline phosphatase was lower after synbiotics; however, a statistically significant difference was observed only for Syn2. The activity of gamma glutamyl transferase was also lower after synbiotics but, in turn, a statistically significant change was observed after Syn1 administration (Table 4).

**Table 3:** Hormone and metabolic profiles in blood serum

Items	Control	Synbiotic 1	Synbiotic 2
Insulin, nmol/L	0.184 ± 0.027	0.167 ± 0.032	0.251 ± 0.058
Glucagon, nmol/L	0.088 ± 0.010	0.091 ± 0.019	0.087 ± 0.020
Insulin/Glucagon, mol/mol	2.592 ± 0.812	2.372 ± 0.699	3.459 ± 0.770
Corticosterone, ng/mL	96.21 ± 3.10	101.7 ± 11.34	97.62 ± 6.01
Leptin, ng/mL	0.170 ± 0.047	0.139 ± 0.012	0.161 ± 0.019
Total T4, ng/mL	19.35 ± 1.59	16.75 ± 0.75	16.99 ± 1.08
Free T4, pg/mL	8.827 ± 1.703	10.350 ± 1.071	8.575 ± 1.110
Total T3, ng/mL	0.774 ± 0.150	0.971 ± 0.227	0.761 ± 0.164
Free T3, pg/mL	1.847 ± 0.126	2.270 ± 0.272	1.684 ± 0.132
Glucose, mg/dL	206.40 ± 1.51	201.91 ± 2.53	198.48 ± 5.34
NEFA, mmol/L	0.56 ± 0.02	0.68 ± 0.03	0.56 ± 0.02
Triglycerides, mg/dL	115.61 ± 11.19	124.20 ± 8.71	106.62 ± 8.36
Total cholesterol, mg/dL	121.53 ± 2.28	117.95 ± 9.46	119.28 ± 6.57
Free cholesterol, mg/dL	10.06 ± 0.97	10.57 ± 3.32	9.53 ± 1.66

Results are means ± SEM (*n* = 8)

**Table 4:** Activity of transferases and alkaline phosphatase in the blood serum

Target, IU/L	Control	Synbiotic 1	Synbiotic 2
Alanine aminotransferase (ALT)	7.04 ± 1.49	6.63 ± 0.90	5.53 ± 0.75
Aspartate aminotransferase (AST)	68.78 ± 6.89	76.73 ± 3.86	67.98 ± 5.54
Alkaline Phosphatase (ALP)	519.6 ± 34.96	394.9 ± 72.07	355.5* ± 50.26
Gamma glutamyl transferase (GGT)	4.423 ± 0.807	2.371* ± 0.507	2.844 ± 0.408

Results are means ± SEM (n = 8). *P < 0.05, compared with control

## Discussion

The heightened activity of all enzymes in the pancreases may be ascribed to their greater stored amount in exocrine cells. Thus, by elevating trypsin activity the synbiotics prepared organisms especially well to digest proteins. The most effective was Syn2, which increased 2.5-fold the “potential” activity of pancreases and 1.5-fold trypsin activity in the content of duodenum. Also, none of the in ovo injected synbiotics decreased the amylolytic or lipolytic potential of pancreases. The changes observed after in ovo administration of synbiotics are not unusual, because alterations in the total activity of digestive enzymes in the pancreas had been observed previously by Pruszynska-Oszmalek et al. [1]. In that paper, in ovo injected synbiotics (inulin + *Lactococcus lactis* spp. *lactis*; Bi^2^tos + *Lactococcus lactis* subsp. *cremoris*) elevated the amylolytic and lipolytic activity of the pancreas at the end of the rearing period, and synbiotics based on Bi^2^tos also elevated trypsin activity. In the present paper, we confirmed that synbiotics injected early during development may exert long-lasting effects on pancreatic enzymes. Additionally, the present study also covers the activity of the main pancreatic enzymes in the duodenal content, which reflects their ability to digest three main groups of nutrients: proteins, lipids and carbohydrates. Elevated activities of trypsin and lipase indicate that Syn2 may improve the process of protein and lipid digestion. However, the amylolytic activity in the intestine was strongly decreased after synbiotic treatment. This phenomenon was probably the result of a lower level of secretion of amylase to the duodenum. It is possible that, as a result, the concentration of glucose in the duodenum content was lowered. In comparison to control birds, appropriate averages were 88.7% and 90.4% after treatment with Syn1 and Syn2, respectively; both changes were statistically significant at *P* < 0.05. This, in turn, was reflected in a lower secretion of incretins to the blood. This could offer an explanation for the parallel decrease in amylase activity in the gastrointestinal tract, diminished expression of mRNA for incretins and their lowered concentration in the blood serum. Additionally, the increase in trypsin and lipase activity could be connected with the lower levels of GLP-1 after synbiotic application. Intravenous infusion of GLP-1 reduced trypsin and lipase activity in men [28]. Simultaneously, none of the effects of synbiotics on amylase content in pancreas could be explained by the fact that GLP-1 does not mediate amylase release from a model of pancreatic cells (AR42J), as observed previously [29]. Similarly, no effect of GIP on amylase release from dispersed pancreatic acinar cells was noted by Sjodin and Conlon [30]. On the other hand, it is difficult to explain the strongly diminished amylolytic activity in the duodenum content from the above observations. It seems to be the case that secretion of amylase to the duodenum is also controlled by other mechanisms. Nevertheless, incretins appear to be an important element in the duodeno-pancreatic and duodeno-brain loops. Besides the continuously explored new functions of incretins, they are important for such key processes as insulin secretion and appetite regulation. Honda reviewed and discussed a role of some incretins in food intake regulation [31]. Turton et al. described the presence of GLP-1 and its receptors in the hypothalamus and the suppressive effect of GLP-1 given ICV on food intake in rats [32]. Also, the same effect has been noted in chicken [33]. The two incretins, GLP-1 and GIP, are secreted by L and K cells of the intestine, respectively. These cells are believed to be sensors of the nutrient ingredients passing through the intestine [15]. Of these, glucose strongly stimulates both GLP-1 release [15] and GIP secretion.

In the present paper, the mRNA transcripts of both investigated incretins were strongly reduced in the duodenum and these changes were accompanied by a very highly diminished concentration of GLP-1 and a significantly reduced level of GIP in the blood serum. Simultaneously, expression of mRNA for both receptors in the pancreases was lower and for GLP-1R this was statistically significant. This situation should not promote insulin secretion by meal via incretins. However, no significant changes were observed either for insulin level, which was not lower in the blood serum after synbiotics, or for other investigated hormones: glucagon, corticosterone, leptin. Also, metabolic parameters, glucose, non-esterified acids, triglycerides, cholesterol, total proteins and albumins in the blood serum were unchanged following in ovo treatment with synbiotics. Thus, the alterations in the activity of pancreatic enzymes and in incretins did not translate into changes in additional parameters, such as other hormones and biochemical indices. Also, in parallel studies within this experiment a lack of synbiotic-induced changes on breeding parameters was observed, as calculated for the whole period from days 1 to 41 [25]. Neither body weight gain nor feed intake, feed conversion efficiency or mortality differed significantly between control and experimental groups treated with synbiotics.

In the present study, a multilateral network of connections was observed, involving the synbiotic – incretins – pancreatic digestive potential – activity of enzymes in duodenum. Synbiotics given in ovo initiate changes and are the first link in the chain of events finally exerting long-lasting effects. The succession of subsequent changes can be differently interpreted. On the one side, the altered microbiome could in no way reduce the amylolytic activity in duodenum by restriction of amylase secretion. In turn, the slowed down digestion of complex carbohydrates and smaller amount of glucose might lower expression of incretins in the duodenum and their level in the blood. On the other hand, alterations within incretins could precede changes in enzyme activity in the pancreas and in the duodenum. Whatever the sequence of events, it is undeniable that the activities of digestive enzymes in the pancreas and in the duodenum are associated with synbiotic in ovo delivery, incretin expression and their concentration in the blood. It is an open question as to whether the noticed effect on both incretin and enzyme activity is a general property of various synbiotics or is just characteristic for those chosen. These results showed that synbiotics are able to change trypsin, amylase and lipase activity in the chicken duodenum. Previous studies performed on humans and rodents have suggested that modulation of gut microbiota by synbiotics or probiotics can affect incretin expression [4, 34]. However, our results are the first to link a few elements: in ovo technique, synbiotics, incretins and digestive enzymes in broiler chickens. A search of the literature provides limited information about the role of incretins in chicken physiology. It had been shown previously that intracerebroventricular injection of GLP-1 inhibits food intake in neonatal chicks [33, 35]. Additionally, administration of GLP-1 increased corticosterone in blood serum [35]. In contrast, in our studies no corticosterone changes were stated. Furthermore, the observed alterations in the pancreatic duodenal axis and expression of incretins did not transpose onto breeding parameters. The organisms of the birds probably adapted themselves to a new physiological situation by switching on/off other mechanisms. On the other hand, it is significant that the synbiotics did not lead to a deterioration in rearing parameters.

Together with analyzing enzymatic, hormonal, and biochemical indices linked directly to body growth and metabolism, diagnostic enzyme activity in the blood serum was also measured to estimate the overall health of broilers. ALP and GGT were used as markers of bone diseases (ALP) and liver and bile duct diseases (GGT and ALP). While not statistically heightened, in two cases lower values of ALP (Syn2) and GGT (Syn1) indicated a good or even improved health status for synbiotic-treated birds. Other health markers are aminotransferases. Their activity increases in disorders such as liver damage. The activity of aminotransferases in the blood serum was not significantly changed, which indicates no negative action from the used synbiotics on the health status of birds. Also, Pruszynska-Oszmalek et al. [1] stated that in ovo administered synbiotics (inulin plus *Lactococcus lactis* subsp. *lactis*; Bi^2^tos plus *Lactococcus lactis* subsp. *cremoris*) had no negative effect on the activity of alanine and aspartate aminotransferases. However, on the contrary, the activity of both aminotransferases was diminished. Also, after treatment with prebiotic Bi^2^tos alanine aminotransferase activity was significantly lower. The activity of alanine and aspartate aminotransferases has also been measured by other authors. Salarmoini and Fooladi [36] investigated an addition of probiotic *L. acidophilus* and did not note any elevation of the activities of aminotransferases and so no negative consequences of the use of these bacteria. Also, *Saccharomyces cerevisiae* used as probiotics which had a beneficial effect on body weight and feed consumption efficiency in chickens did not elevate the activities of aminotransferases [37]. Analyzing and summarizing data obtained on the influence of the two investigated synbiotics (*Lb. salivarius* IBB3154 plus galactooligosaccharide [Bi^2^tos] and *Lb. plantarum* IBB3036 plus raffinose family oligosaccharides [RFO]), it seems to be obvious that they may be used as in ovo additives in chickens without negative, up to the 6^th^ week, effects on health and basic production parameters. Simultaneously, they must affect the pancreas, evoking significant alterations in the activity of gastrointestinal tract digestive enzymes and in the expression of incretins. So, they influence an important function of the organism, which is proper food digestion, and they downregulate (control) expression of incretins. As mentioned above, synbiotics and prebiotics can modulate intestinal microflora. Based on these findings, literature data indicate a few possible pathways by which probiotics and synbiotics can affect secretion and expression of incretins in K- and L-cells. The most convincing seems to be the effect of microbiomes on short chain fatty acid (SCFA) concentrations in the intestine. SCFA are produced during bacterial fermentation of dietary fiber or of non-absorbed carbohydrate. It was shown in the literature that there exists a link between fiber, gut microbiota and K, L-cells acting via GPR41, GPR43, FFAR2 and FFAR3. Moreover, a possible role of FFAR2 in GLP-1 secretion was suggested by the discoveries that SCFA temporary triggered Ca^2+^ in primary L-cells and mice lacking *ffar*2 or *ffar*3 exhibited reduced SCFA-triggered GLP-1 secretion in vitro [38, 39]. The mechanisms which combine the incretins with the exocrine pancreas and digestion in the intestine of chickens require further investigation and the fact should be considered that the results obtained for specific synbiotics in broiler chickens should not be generalized to other synbiotics, other populations of chicken as well as other poultry and livestock species and, of course, humans.

## Conclusions

In summary, we found that in ovo injection of two different synbiotics [*Lb. salivarius* plus galactooligosaccharide (Syn1) and *Lb. plantarum* plus RFO (Syn2)] leads to a lowering of the level of incretins (GLP-1 and GIP) in blood serum. Furthermore, these two synbiotics downregulated *GLP-1* and *GIP* mRNA expression in the duodenum and GLP-1R in the pancreas. Moreover, Syn2 increased trypsin and lipase activities in the duodenum content, simultaneously decreasing amylase activity. Such activity can promote digestion of proteins and lipids. Our results suggest that synbiotics can be directly or indirectly involved in incretin secretion and reception.

## Abbreviations

ALP: alkaline phosphatase
ALT: alanine aminotransferase
AST: aspartate aminotransferase
GGT: gamma glutamyl transferase
GIP: gastric inhibitory polypeptide or glucose-dependent insulinotropic peptide
GIP-R: gastric inhibitory peptide receptor
GLP-1: glucagon like peptide 1
GLP-1R: glucagon like peptide 1 receptor
RFO: raffinose family oligosaccharides
Syn1: synbiotic 1 (*Lb. salivarius* IBB3154 plus galactooligosaccharide – Bi^2^tos)
Syn2: synbiotic 2 (*Lb. plantarum* IBB3036 plus RFO)
